# Pemphigus végétant: à propos d'un cas

**DOI:** 10.11604/pamj.2013.14.101.2478

**Published:** 2013-03-13

**Authors:** Sanaa Lemtibbet, Badreddine Hassam

**Affiliations:** 1 Service de Dermatologie, CHU Ibn Sina, Rabat, Maroc

**Keywords:** Pemphigus végétant, pemphigus vulgaire, lésion cutanée, IgG, vegetating pemphigus, pemphigus vulgaris, skin lesion, IgG

## Image en médecine

Le pemphigus végétant est considéré comme une forme rare du pemphigus vulgaire, caractérisée par une atteinte des plis et un aspect végétant des lésions par endroit. Nous rapportons le cas d'un patient de 28 ans qui présentait depuis 2 mois des lésions érosives et bulleuses du gland et de la région inguinale, prenant un aspect végétant et augmentant progressivement de taille avec apparition par la suite de nouvelles lésions intéressant les creux axillaires et le tronc associé à une conjonctivite, une chéilite et des érosions endobuccales. Une biopsie cutanée réalisée objectivait un clivage suprabasale avec acantholyse, associés à une papillomatose intense aboutissant à la formation de végétations. L'immunofluorescence directe montrait un dépôt d'IgG à la surface des kératinocytes en “maille de filet” et enfin l'immunofluorescence indirecte révélait la présence d'anticorps anti-substance intercellulaire positifs à 640 UI/ml, confirmant ainsi le diagnostic de pemphigus végétant. Sur le plan thérapeutique, on a mis le patient sous corticothérapie générale: Prédnisone à raison de 2mg/kg/j associée à des soins locaux. L’évolution était marquée par une rémission complète au bout de 2 mois d'où la dégression progressive de la corticothérapie de 10% de la dose totale tous les 10 jours sans rechute.

**Figure 1:**
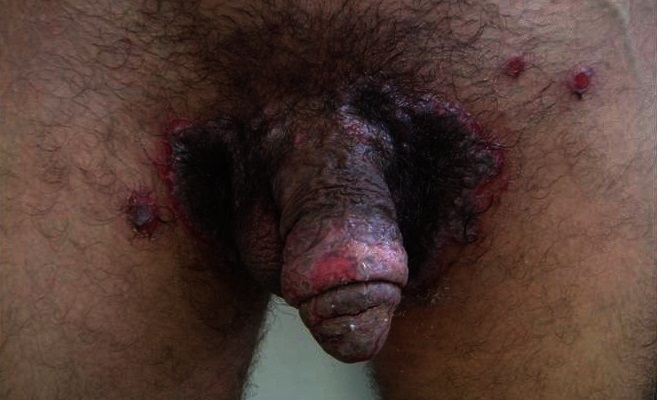
Placard végétant, papillomateux, suintant et fétide de la région inguinale associé à des érosions au niveau du gland

